# Experimental Infection of Domestic Pigs with an African Swine Fever Virus Field Strain Isolated in 2021 from the Dominican Republic

**DOI:** 10.3390/v14051090

**Published:** 2022-05-19

**Authors:** Elizabeth Ramirez-Medina, Vivian O’Donnell, Ediane Silva, Nallely Espinoza, Lauro Velazquez-Salinas, Karen Moran, Dee Ann Daite, Roger Barrette, Bonto Faburay, Robin Holland, Douglas P. Gladue, Manuel V. Borca

**Affiliations:** 1 Plum Island Animal Disease Center, Agricultural Research Service, USDA, Greenport, NY 11944, USA; elizabeth.ramirez@usda.gov (E.R.-M.); ediane.silva@usda.gov (E.S.); nallely.espinoza@usda.gov (N.E.); lauro.velazquez@usda.gov (L.V.-S.); 2 Plum Island Animal Disease Center, Animal and Plant Health Inspection Service, USDA, Greenport, NY 11944, USA; vivian.odonnell@usda.gov (V.O.); karen.moran@usda.gov (K.M.); deeann.daite@usda.gov (D.A.D.); roger.barrette@usda.gov (R.B.); bonto.faburay@usda.gov (B.F.); robin.holland@usda.gov (R.H.)

**Keywords:** ASFV, ASF, Dominican Republic, virulence

## Abstract

African swine fever virus (ASFV) is the etiological agent of African swine fever (ASF), a disease of domestic and wild swine that has spread throughout a large geographical area including Central Europe, East and Southeast Asia, and Southern Africa. Typically, the clinical presentation of the disease in affected swine heavily depends on the virulence of the ASFV strain. Very recently, ASFV was detected in the Dominican Republic (DR) and Haiti, constituting the first diagnosis of ASFV in more than 40 years in the Western hemisphere. In this report, the clinical presentation of the disease in domestic pigs inoculated with an ASFV field strain isolated from samples collected in the DR (ASFV-DR21) was observed. Two groups of domestic pigs were inoculated either intramuscularly (IM) or oronasally (ON) with ASFV-DR21 (10^4^ hemadsorbing dose-50% (HAD_50_)). A group of naïve pigs (designated as the contact group) was co-housed with the ASFV-DR21 IM-inoculated animals to evaluate ASFV transmission and disease manifestation. Animals inoculated IM with ASFV-DR21 developed an acute disease leading to humane euthanasia at approximately day 7 post-inoculation (pi). Interestingly, animals inoculated via the ON route with ASFV-DR21 developed a heterogeneous pattern of disease kinetics. One animal developed an acute form of the disease and was euthanized on day 7 pi, another animal experienced a protracted presentation of the disease with euthanasia by day 16 pi, and the remaining two animals presented a milder form of the disease, surviving through the 28-day observational period. The contact animals also presented with a heterogenous presentation of the disease. Three of the animals presented protracted but severe forms of the disease being euthanized at days 14, 15 and 21 pi. The other two animals presented with a milder form of the disease, surviving the entire observational period. In general, virus titers in the blood of animals in all study groups closely followed the clinical presentation of the disease, both in length and extent. Importantly, all animals presenting with a prolonged form of the disease, as well as those surviving throughout the observational period, developed a strong ASFV-specific antibody response. These results suggest that ASFV-DR21, unless inoculated parenterally, produces a spectrum of clinical disease, with some animals experiencing an acute fatal form while others presented with a mild transient disease accompanied by the induction of a strong antibody response. At the time of publication, this is the first report characterizing the virulent phenotype of an ASFV field strain isolated from samples collected in the DR during the 2021 outbreak and provides information that may be used in developing epidemiological management measures to control ASF on the island of Hispaniola.

## 1. Introduction

African swine fever (ASF) is a high consequence infectious disease affecting domestic and wild swine with variable clinical presentation depending on the strain of ASF virus [1]. After its initial emergence, ASF was largely restricted to the continent of Africa, but after an outbreak occurred in the Republic of Georgia in 2007, the disease spread through the commercial swine industry and feral swine from Central Europe to Southeast Asia. Recently, ASF was confirmed in the Dominican Republic (DR), the first detection of ASF after more than 40 years in the Western hemisphere. The etiological agent of ASF, ASF virus (ASFV), is a structurally complex virus harboring a large double-stranded DNA genome that encodes for more than 150 genes [2] and has been classified into more than 24 genotypes [1]. The genetic heterogeneity is suggested to be the basis of different virulent phenotypes, both between genotypes and within genotypes, observed in infected domestic swine.

At the time of publication, no commercial vaccine is available to prevent ASF; therefore, disease management in ASF-infected areas predominately relies on culling infected animals and restricting the transport of susceptible animals and animal products [2]. By understanding the clinical characteristics of ASFV-infected animals and the transmissibility of the disease, management strategies to control the disease may be effectively developed and implemented. 

Since the detection of ASF in the DR in 2021, the disease has spread to nearly all provinces in the country and has also been detected in the neighboring country of Haiti. ASF has potential serious economic implications for the swine industry in the DR and requires the implementation of epidemiological measures to prevent, control and potentially eradicate the disease. In this report, the clinical presentation of domestic pigs inoculated with an ASFV strain isolated from samples collected in the DR (ASFV-DR21) was evaluated. 

## 2. Materials and Methods 

### 2.1. Cell Culture and Preparation of Virus Inoculum

Peripheral blood mononuclear cells (PBMC) were prepared from fresh defibrinated swine blood as previously described [3]. After preparation, adherent cells were seeded at 150 × 10^6^ per Primaria T75 cm^2^ flask (Corning, Corning, NY, USA) and incubated at 37 °C in a humidified 5% CO_2_ incubator for 3–4 days before infection. Virus titration was performed on primary swine macrophage cell cultures in 96-well plates. Virus dilutions and cultures were performed using macrophage medium [3]. The presence of virus was assessed by hemadsorption (HA), and virus titers were calculated by the Reed and Muench method [4]. As performed, detection of infectious virus has a sensitivity of ≥1.8 HAD_50_/mL.

The DR21 ASFV isolate was recovered from a pig in the province of Espaillat, in the Dominican Republic, on July 2021. The isolate belongs to genotype II based on characterization of the p72 sequence showing 100% identity with Georgia 2007 in the pairwise alignment. The isolate accumulated 11 SNPs since sharing a common ancestor with GA 2007. Additionally, comparing the SNPs alone across the whole genome, the DR isolate is 99.99% identical to Georgia 2007. ASFV-DR21 virus inoculum was prepared from a whole blood sample that originated in the Dominican Republic from a pig that tested positive by PCR for ASFV-DR21. The original, field-collected whole blood sample was used to inoculate primary swine macrophage cell cultures in Primaria T25 cm^2^ flasks. The flask was monitored for the development of viral cytopathic effect (CPE), as compared with a mock infected flask. At 90% CPE, supernatant was harvested, aliquoted and store at −70 °C for further characterization. Virus titration was performed on primary swine macrophage cell cultures as describe above. ASFV-DR21 stock genome sequencing was completed on an Illumina MiSeq System using the Nextera XT library preparation kit and the 500-cycle v2 sequencing kit (Illumina; San Diego, CA, USA), to rule out the presence of any extraneous agents and to confirm that the ASFV DR21 belongs to the ASFV genotype II aligning with the Georgia 2007 virus (data not shown), as previously described in the OIE report (https://wahis.oie.int/#/report-info?reportId=37535). 

### 2.2. Quantitative Real-Time PCR Protocol for the Detection of ASFV DNA 

To assess the presence of ASFV in oral and nasal swabs [5] at different time points during the study, real-time PCR (qPCR) was conducted to detect the *B646L* gene (encoding for p72 protein) of ASFV as previously described [6]. Briefly, viral DNA was extracted using the MagMAX™ Pathogen RNA/DNA kit (ThermoFisher Scientific, Waltham, MA, USA) on a KingFisher Flex automated extraction and purification system (ThermoFisher Scientific, Waltham, MA, USA). Master mix was prepared using the TaqMan™ Universal PCR Master Mix (Applied Biosystems, Waltham, MA, USA), and the following primers forward: 5′-CTTCGGCGAGCGCTTTATCAC-3′, reverse: 5′-GGAAATTCATTCACCAAATCCTT-3′ and probe: 5′-FAM-CGATGCAAGCTTTAT-MGB NFQ-3′. Assays were conducted on an Applied Biosystems 7500 Real-time PCR system. As performed, the sensitivity of detection is 1.28 DNA copies/reaction volume and 10^2.55^ HAD50/mL [6].

### 2.3. Animal Infections

ASFV-DR21 virulence phenotype was assessed using 80–90 pound commercial Yorkshire crossbreed female swine [3]. Five pigs were inoculated intramuscularly (IM) with 1 mL of DMEM (Gibco, Waltham, MA, USA) containing 10^4^ tissue culture infectious dose (HAD_50_) of ASFV-DR21. A second group of five pigs was oronasally (ON) inoculated by instilling in each animal 1 mL containing 10^4^ HAD_50_ of ASFV-DR21 intranasally and another 1 mL of an equal concentration of virus via oral administration. A third group of five uninoculated pigs was co-housed with the IM-inoculated animals 24 h after inoculation to represent a co-housed viral transmission group. Animals from the IM-inoculated and uninoculated groups shared food and water in a single room with a floor surface of 110 square feet throughout the duration of the study. Clinical signs (anorexia, depression, fever, purple skin discoloration, staggering gait, diarrhea, and cough) and changes in body (rectal) temperature were recorded daily throughout the study period of 28 days. Clinical samples (blood, serum, nasal swabs, and oral swabs) were collected on days 2, 4, 6, 8, 10, 12, 14, 19, 22, 25 and 28 post-inoculation for detecting the presence of the virus by qPCR and virus titrations in swine macrophage cultures, as earlier described, and ASFV-specific antibodies by an enzyme-linked immunosorbent assay (ELISA) as described below. 

Animal infection studies were performed under biosafety level 3-agriculture conditions at the Plum Island Animal Disease Center (PIADC) animal facility following protocols approved by the PIADC Institutional Animal Care and Use Committee (IACUC) of the US Departments of Agriculture and Homeland Security (protocol number 225.06-19-R, approved 09-10-19).

### 2.4. Detection of Anti-ASFV Antibodies

ASFV antibody detection was performed using an in-house ELISA as described previously [7]. Briefly, ELISA antigen was prepared from Vero cells infected with a Vero adapted strain of ASFV Georgia 2010 isolate. Maxisorb ELISA plates (Nunc, St. Louis, MO, USA) were coated with 1 µg per well of infected or uninfected cell extract. The plates were blocked with phosphate buffered saline containing 10% skim milk (Merck, Kenilworth, NJ, USA) and 5% normal goat serum (Sigma, Saint Louis, MO, USA). Each swine serum sample was tested at multiple dilutions against both infected and uninfected cellular antigen. ASFV-specific antibodies in the swine sera were detected using an anti-swine IgG-horseradish peroxidase conjugate (KPL, Gaithersburg, MD, USA) and SureBlue Reserve peroxidase substrate (KPL). Plates were read at OD630 nm in an ELx808 plate reader (BioTek, Shoreline, WA, USA). Antibody titers were expressed as the log_10_ of the highest dilution where the OD630 nm reading of the tested sera at least duplicated the reading of the mock infected (obtained at day 0 post infection) sera. Confirmation of antibodies to ASFV was also evaluated by use of a commercially available blocking ELISA for antibodies to the structural protein VP73 of ASFV (Ingenasa INgenzim PPA COMPAC ASF ELISA Kit, Prionics, Zurich, Switzerland) following recommended manufacturer’s procedures. OD was measured at 450 nm using a plate reader (Molecular Devices, VersaMax kinetic plate reader) and data acquired (Molecular Devices, SoftMAX PRO, San Jose, CA, USA).

## 3. Results

### 3.1. Clinical Disease in Animals Inoculated with ASFV-DR21

This study was performed to evaluate the virulence phenotype of the ASFV-DR21 strain under laboratory-controlled conditions using different routes of infection. The study was composed of three animal groups: one group inoculated IM with 10^4^ HAD_50_ per animal, the second group inoculated ON by instillation with 2 × 10^4^ HAD_50_ total per animal (10^4^ HAD_50_ nasally and orally per animal), and a third group composed of uninoculated naïve animals serving as contact animals cohabitating with the group of animals IM inoculated beginning at 24 h pi.

**Figure 1:**
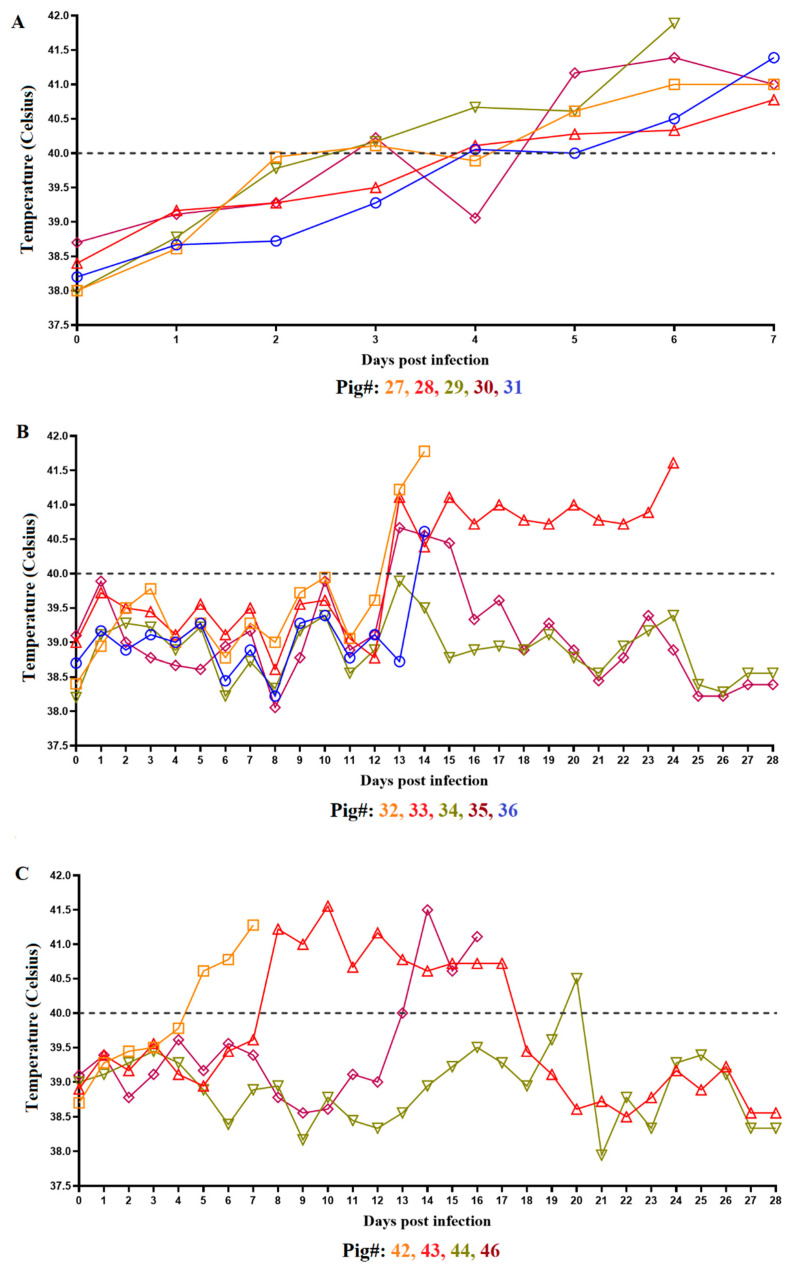
Body temperature measurements from animals inoculated with ASFV-DR21 IM (**A**), by contact (**B**), or ON (**C**) from day 1–28 pi. Data are presented as individual values (expressed as °C), with different colored lines and symbols representing individual animals. The horizontal dashed line denotes 40 °C. Animal #45 was excluded from the data presented due to removal from the study on day 0.

**Figure 2:**
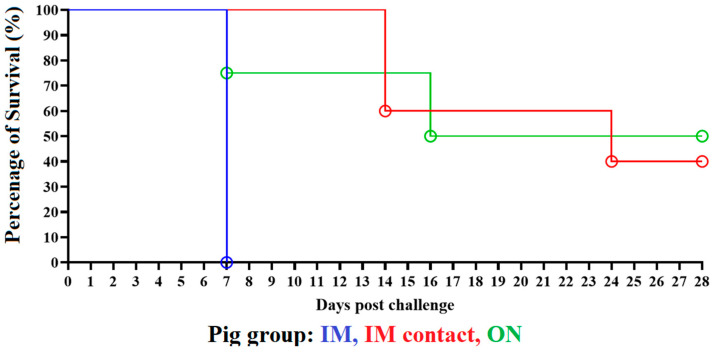
Kaplan-Meier survival curve of animals inoculated with ASFV-DR21 IM (blue), ON (green), or by contact (red) from day 1–28 pi. Data are presented as percentage of survival of animals within a study group.

The group of five animals IM inoculated with ASFV-DR21 (animal ID’s #27–31) began developing an increased body temperature by day 3 pi, with temperatures surpassing 40 °C (104 °F) and all animals maintaining ≥40 °C temperatures by day 5 pi. The elevation of body temperature was accompanied by the development of clinical signs consistent with ASF, including depression, anorexia, and red-purple coloration in the skin, which progressed to a severe form of the disease. All animals in this group reached clinical endpoint due to disease progression, requiring humane euthanasia by day 6 or 7 pi (Figure 1A and Figure 2).

Animals inoculated ON with ASFV-DR21 (animal ID’s #42–46) presented with varied phenotypes compared to the IM-inoculated animals. Out of the five inoculated animals, one animal (#45) was euthanized at day 0 pi due to a methodological error unrelated to the viral infection. Animal #42 rapidly developed an acute form of ASF and was clinically undistinguishable from the animals inoculated IM with ASFV-DR21, with humane euthanasia by day 7 pi due to disease progression (Figure 1C and Figure 2). Animal #43 developed an elevated body temperature by day 8 pi, surpassing and maintaining > 40 °C body temperature until day 18 pi, when the body temperature dropped and was maintained within normal limits until the completion of the study at 28 pi. The observed clinical disease in animal #43 aligned with measured trends in body temperature, with the observation of lethargy, decreased appetite, and skin discoloration that gradually resolved during the last 10 days of observation. By day 28 pi, this animal was clinically normal. Animal #46 remained clinically normal through the initial phases of the study, with body temperature measurements within normal limits, until a rapid rise of body temperature at day 13 pi accompanied by clinical disease, ultimately reaching clinical endpoint and humane euthanasia at day 16 pi. Lastly, animal #44 did not develop clinical disease and maintained body temperature measurements within normal limits throughout the duration of the study, aside from an isolated measurement of >40 °C on day 20 pi.

The naïve animals cohabitating with the ASFV-DR21 IM-inoculated group (animal ID’s #32–36) also presented a variable phenotype of clinical observations. Animals #32 and 36 remained clinically normal until day 12 and 13 pi, respectively, when a rapid rise in body temperature (>40 °C) accompanied by progressive and severe clinical disease were observed, resulting in clinical endpoint with humane euthanasia on day 14 pi (Figure 1B and Figure 2). Similarly, animal #33 also developed a rapid elevation of body temperature, surpassing and maintaining > 40 °C, at day 13 pi, but with a milder form of the disease that gradually worsened before reaching clinical endpoint and humane euthanasia by day 24 pi. Animals #34 and 35 were clinically normal throughout the duration of this study, with the exception of a moderate increase in body temperature (surpassing 40 °C) in animal #35 during days 13–15 pi. 

### 3.2. Evaluation of Virus Replication and Shedding in Animals Inoculated with ASFV-DR21 and Transmission to Naïve Animals

The kinetics of virus replication and virus secretion were evaluated in all three animal groups, including both ASFV-DR21-inoculated groups and the contact animal groups, by determining viremia titers at different sampling times. 

**Figure 3:**
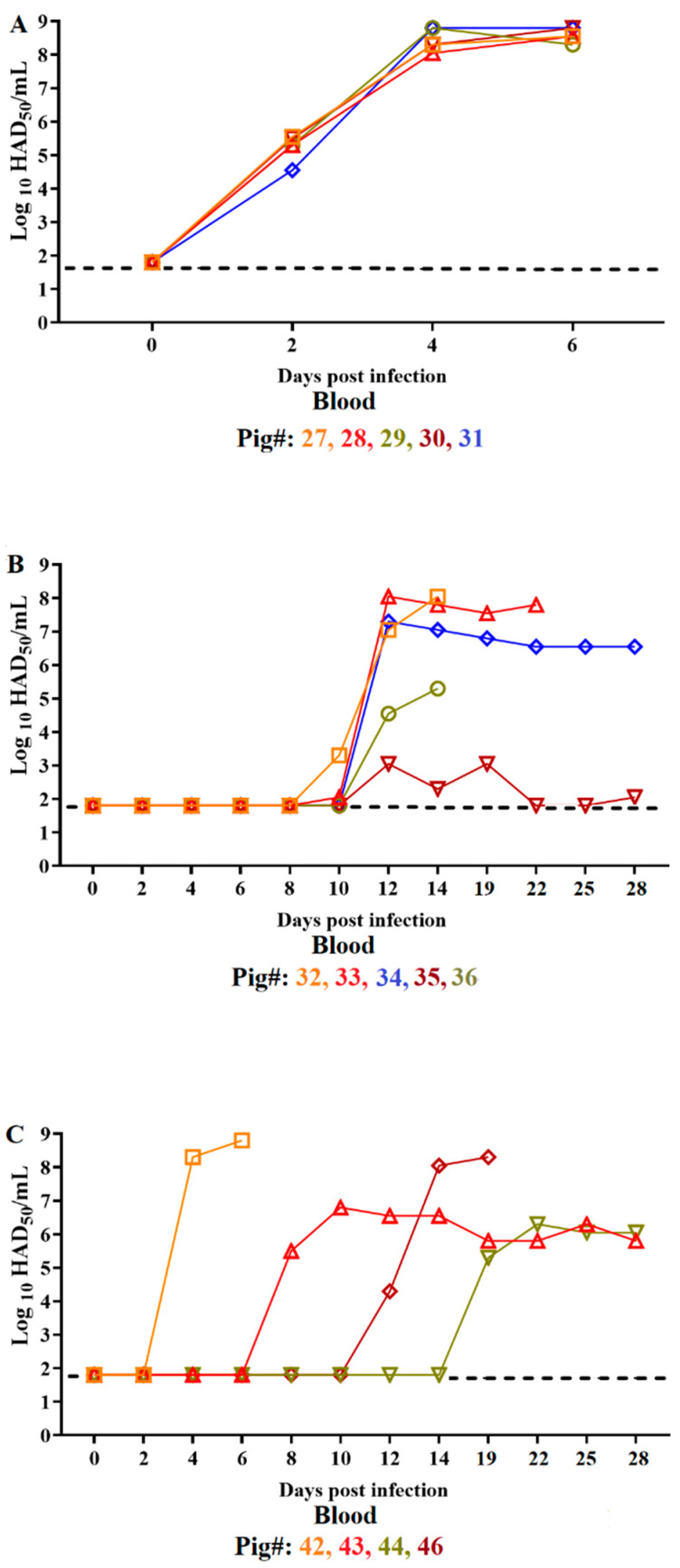
Virus titers in blood collected from animals inoculated with ASFV-DR21 IM (**A**), by contact (**B**), or ON (**C**) from day 1–28 pi. Data are presented as individual values of virus titers (expressed as HAD_50_/mL), with different colored lines and symbols representing individual animals. Sensitivity of detection ≥ 1.8 log_10_/mL. Animal #45 was excluded from the data presented due to removal from the study on day 0.

**Figure 4:**
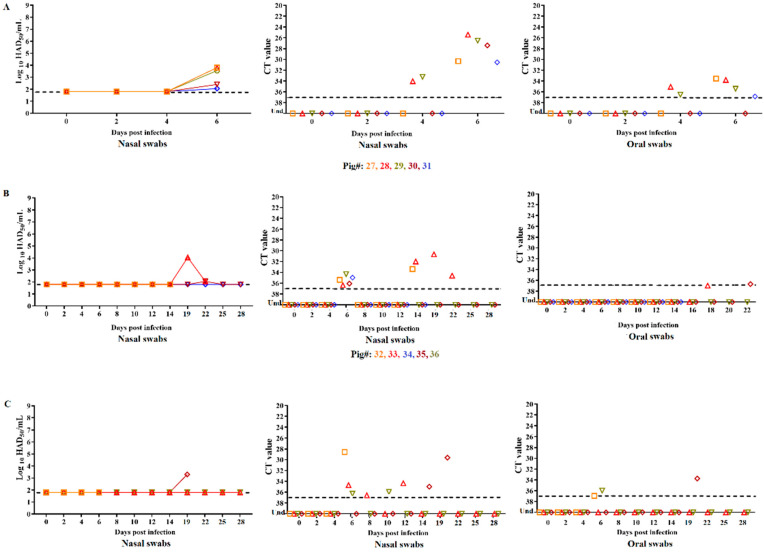
Presence of virus in nasal (detected by titration of infectious virus and qPCR) and oral swabs (detected by qPCR) collected from animals inoculated with ASFV-DR21 IM (**A**), by contact (**B**), or ON (**C**) from day 1–28 pi. Data are presented as individual values of virus titers (expressed as HAD_50_/mL), and qPCR CT values with different colored lines and symbols representing individual animals. Sensitivity of detection of infectious virus is ≥1.8 log_10_/mL and considered positive by qPCR with CT values ≤ 37. Animal #45 was excluded from the data presented due to removal from the study on day 0.

The group of IM-inoculated animals (#27–31) developed moderate viremia titers as early as day 2 pi with values ranging from 10^4.5^ to 10^5.5^ HAD_50_/mL in whole blood samples. ASFV-DR21 viral titers rapidly increased to high viremia values ranging from 10^8.05^ to 10^8.8^ HAD_50_/mL by day 4 pi and maintained that level of viremia until the animals reached clinical endpoint at day 7 pi (Figure 3A). While ASFV infectious virus in nasal swabs remained undetected in samples obtained from all animals at days 2 and 4 pi, all animals developed low to moderate virus titers (10^2.05^ to 10^3.8^ HAD_50_/mL) by day 6 pi. Detection by qPCR demonstrated presence of virus genome since day 4 pi in animals #28 and 29 with all animals becoming positive by day 6 pi. Infectious ASFV was not detected at any time point in oral swabs obtained from animals in this study group, but virus genome was detected in animals #28 and 29 by day 4 pi and in all animals except #30 by day 6 pi (Figure 4A).

Virus replication in animals inoculated ON with ASFV-DR21 closely aligned with the presentation of clinical signs associated with ASF. Therefore, animal #42 presented high viremia titers on days 4 and 6 pi (10^8.3^ to 10^8.8^ HAD_5__0_/mL, respectively) just before reaching humane clinical endpoint. Infectious ASFV-DR21 was not detected in the nasal or oral swabs of animal #42 at any of the tested time points (Figure 4C). Viremias in animal #46 remained undetectable until day 12 pi when 10^4.3^ HAD_50_/mL was detected with a subsequent rapid increase to titers of 10^8.05^ and 10^8.3^ HAD_50_/mL at days 14 and 19 pi, respectively, before reaching humane endpoint. ASFV-DR21 was not detected in any oral swab samples tested and low virus titers (10^3.3^ HAD_50_/mL) were only detected from the final nasal swab sample (day 19 pi). The two animals surviving the entire course of the study developed different viremia kinetics. Viremia in animal #43 was first detected at day 8 pi, with a titer of 10^5.5^ HAD_50_/mL, followed by a persistent viremia with similar titer values until the last day of the infection study. Animal #44 presented a similar pattern of viremia with the difference being undetectable viremia until day 19 pi. Infectious virus was not detected in any nasal or oral swabs with the exception of very low titers in the nasal swabs of animal #46 at day 19 pi.

Virus genome was detected by qPCR, at low levels and intermittently, in nasal swabs of all animals in this group between day 6 and 19 pi. In addition, virus genome was detected by qPCR in oral swabs only on day 6 pi in animals #42 and 44 and on day 19 pi in animal #46 (Figure 4C).

Animals cohabitating with the group of the IM-inoculated pigs also presented a heterogenous pattern of viremia (Figure 3B). Viremia in animals #32 and 36 remained undetected until days 10 and 12 pi, respectively, at which point moderate viral titer values (10^3.3^ and 10^4.55^ HAD_50_/mL, respectively) were detected. Titers increased (10^8.05^ and 10^5.3^ HAD_5__0_/mL, respectively) in both animals until day 14 pi, when humane clinical endpoint was reached. Viremia was undetectable in animal #33 until day 10 pi when a low viremia was detected (10^2.05^ HAD_50_/mL). Viremia values subsequently increased (10^7.55^ to 10^8.05^ HAD_50_/mL) until the day of euthanasia (day 24 pi). Animals #34 and 35 yielded undetectable viremias until day 12 pi, at which point low (10^2.3^ and 10^3.05^ HAD_50_/mL, respectively) or high (10^6.55^ and 10^7.05^ HAD_50_/mL, respectively) viral titer values were measured until the end of the study period (day 28 pi). With the exception of a viral titer of 10^2.05^ HAD_50_/mL in the nasal swab samples obtained on day 19 pi in animal #33 and on day 22 in animal #36, all other nasal and oral swabs from these animals were negative for virus isolation. Virus genome in nasal swabs was detected at low levels by qPCR in all animals at day 6 pi and in animal #33 between days 14 and 22 pi. Oral swabs were all negative by qPCR with the exception of low levels detected in animals #33 and 35 at day 18 and 22 pi, respectively.

### 3.3. Development of an ASFV Antibody Response in Animals Infected with ASFV-DR21

The development of virus-specific antibody responses was evaluated in both ASFV-DR21 inoculated and contact animals surviving the acute form of the disease. Detection of the serological virus-specific antibody response was performed using an in-house ELISA [7] and a commercial ELISA (Ingenasa INgenzim PPA COMPAC ASF ELISA Kit, Prionics, Zurich, Switzerland). 

All animals infected with ASFV-DR21 that were euthanized prior to day 7 pi due to humane clinical endpoint were excluded from data analysis of serological testing due to insufficient time post-infection to develop a systemic antibody response (data not shown). Similarly, animals within the contact group, including animals #32, #36 and ON-inoculated animal #46, which were euthanized on days 14 and 16, respectively, failed to develop a virus-specific antibody response probably due to insufficient time post-infection to generate detectable antibody titers (data not shown).

**Figure 5:**
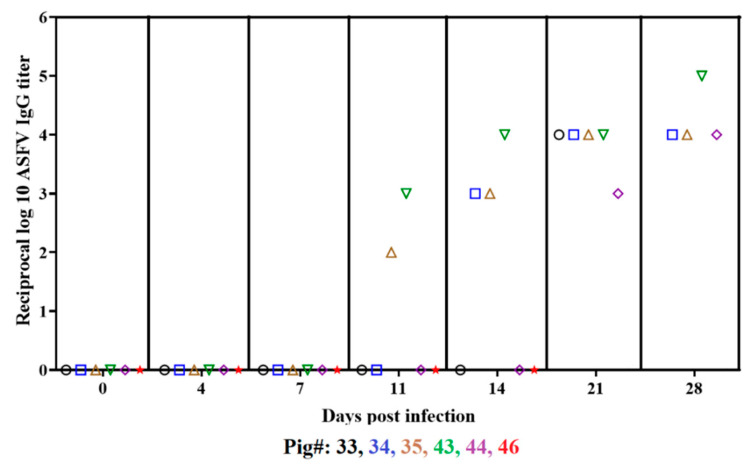
Serological detection of virus-specific antibody titers in the serum of animals surviving infection after inoculation with ASFV-DR21 ON or by contact with IM inoculated animals. Data are presented as individual values of antibody titers detected by ELISA and expressed as the reciprocal of the log_10_ of the highest dilution where the OD630 nm reading of the tested sera at least duplicated the reading of the mock infected (obtained at day 0 post infection) sera. ASFV IgG titers with different colored symbols representing individual animals.

All animals surviving through the 28 days after infection or housed in close contact developed an ASF virus-specific antibody response (Figure 5). ASFV-DR21 ON-inoculated animals #43 and 44 started developing a measurable antibody response by days 11 and 21 pi, respectively, 2 to 3 days after the first time point with detectable viremia occurred in these animals (8 and 19 days pi, respectively) (Figure 3). Contact animals #35, 34 and 33 developed a detectable antibody response by days 11, 14 and 21 pi, respectively, all with detectable viral titers by day 12 pi (Figure 3). In all animals with detectable serological response, by day 21 or 28, virus-specific antibody titers reached values of at least 10^4^/mL (Figure 4A). Detection of antibody by the commercial ELISA competitive test confirmed results in the ON inoculated and the contact groups and demonstrated absence of detectable response in IM inoculated animals at 7 days pi (data not shown).

## 4. Discussion

The results presented in this study demonstrate that a viral strain isolated from samples collected in the 2021 ASF outbreak in the DR, ASFV-DR21, does not consistently produce an acute and fatal form of the disease unless it is inoculated IM, within a controlled laboratory setting. Animals inoculated with ASFV-DR21 by the oronasal route or by contact with IM-inoculated animals developed a variable pattern of clinical disease with just a proportion of the animals developing an acute and fatal disease. Most of these animals developed mild and prolonged clinical disease and, in some animals, survived with a transient or inapparent form of the disease during the entirety of the study period.

Animals IM-inoculated developed a rapid and ultimately fatal disease, indicating that the virus is able to disseminate systemically (virus was detected as early as day 2 pi) and cause severe clinical disease. In the case of animals comingling with the ASFV-DR21 IM-infected animals, all showed a delayed development of ASF, with viremia detected 8 to 10 days later than in the IM-infected animals. Under the study conditions, the precise time of viral transmission between ASFV-DR21 IM-inoculated animals and naïve contact animals cannot be determined. Given that viral shedding was not detected by nasal swab sampling of the ASFV-DR21 IM-inoculated animals until day 6 pi, it is presumed that viral transmission likely occurred no earlier than day 6 pi. All ASFV-DR21 IM-inoculated animals were euthanized due to clinical endpoint criteria and were thus removed from the animal room by day 7 pi; therefore, since then these animals did not provide a biological source of potentially infectious virus for the contact animals within the cohabitated space. Assuming that the contact animals were infected from nasal secretion of the IM inoculated group by day 5–6 pi, it would mean that development of virus infection in the contact animals took approximately 5 to 7 days from the infection. When the ASFV-DR21 was instilled in the nasal and oral cavity, detectable viremia was delayed when compared to the ASFV-DR21 IM-inoculated group. Given that virus shedding was undetectable or detectable in very low numbers in the ASFV-DR21 ON-inoculated animals, it is presumed that all animals in the group were indeed infected from the virus inoculum, even in animals where viremia was not detected until much later in the study. 

Given the clinical observations and viral shedding data, an association between viral shedding and disease progression is presumed. This observation is clearly seen in all animals inoculated IM with ASFV-DR21 and in the majority of the contact animals cohabitating with the IM-infected animals, where the development of ASF clinical signs oincides with detectable viral titers in the collected clinical samples. 

In addition, the level of infectious virus shedding in nearly all animals was low. Secretion of virus was consistently found, albeit at low-medium titers, in all animals inoculated IM with ASFV-DR21. Conversely, nearly all of the ASFV-DR21 ON-inoculated and contact animals yielded just detectable infectious viral shedding via nasal secretion, although viremia was detected at high titers at some point in the study progression. Given previous observations of oral-nasal secretions of ASFV in infected animals, it was surprising that no shedding of infectious virus could be detected in any of the oral swabs collected from any of the animals throughout the study [5].

These observations, including the low level of virus shedding and the protracted appearance of detectable viremia as well as disease onset in ASFV-DR21 ON-inoculated and contact animals, suggest a decreased ability of ASFV-DR21 to infect and cause disease when inoculated in a controlled laboratory setting via routes of infection that mimic natural transmission. Although the study was specifically designed to evaluate the clinical presentation of ASFV-DR21, there was an observable decrease in virulence under the study conditions performed, especially when subjectively comparing the animals inoculated with ASFV-DR21 via the ON versus IM route. These observations are in agreement with the description of field clinical cases of ASF in DR [8] and in sharp contrast with the virulence demonstrated by the ASFV Georgia 2010 isolate tested under similar experimental conditions. Animals either IM or ON inoculated with even a lower virus dose (10^3^ TCID_50_/mL) present the same disease severity and kinetics, as well produced 100% lethality in all animals regardless the route of inoculation used [9]. 

It was also notable that three animals (#34, 43 and 44) developed a high level of viremia but did not develop a fatal form of the disease. It is possible that this observation is attributable to the presence of an ASFV-specific antibody response that developed in those animals, which may have impacted the disease progression dynamics. These three animals, along with animals #33 and 35, developed strong virus specific antibody responses. The level of those antibody responses is comparable to those elicited by live attenuated experimental vaccines which efficaciously induce protection to vaccinated pigs against the challenge with the homologous parental virus [3,10,11,12,13,14,15]. The presence of an ASFV-specific antibody response in animals surviving the infection with ASFV-DR21 is an important factor to be considered in diagnostic and surveillance strategies to control the ASFV outbreak in the DR.

It should be note that results presented here may varies if animals with different characteristics are used. 

At the time of publication, this is the first report evaluating the clinical presentation of ASFV-DR21, and it also provides observational data on the basic virological dynamics and the serological antibody response in domestic pigs infected under controlled laboratory conditions with ASFV-DR21. These data provide an initial description of ASFV-DR21 virulence, and important information that can be used in disease response measures.

## Data Availability

Not applicable all data is included within the manuscript.
